# A critical appraisal of systematic reviews assessing the effect of chronic velocity-based resistance training on health and athletic performance outcomes: A systematic review

**DOI:** 10.1371/journal.pone.0342992

**Published:** 2026-02-18

**Authors:** Andres F. Loaiza-Betancur, Cristian González-González, Alejandro Díaz-Franco, Jeferson Castaño-Soto, Alejandro Alzate-Toro, Elias Areiza-Usuga, Diego A. Zuluaga-M, Juan Osvaldo Jiménez-Trujillo, Andrés M. Echavarría‑Rodríguez, Víctor Díaz‑López, Iván Chulvi-Medrano, Lisette Ethel Iglesias-González

**Affiliations:** 1 JBI, School of Public Health, Adelaide University, Adelaide, South Australia, Australia; 2 Instituto Universitario de Educación Física y Deporte, Universidad de Antioquia, Medellín, Antioquia, Colombia; 3 Grupo de Investigación en Ciencias de la Actividad Física y el Deporte (GRICAFDE), Universidad de Antioquia, Antioquia, Medellín, Colombia; 4 Research Group in Prevention and Health in Exercise and Sport (PHES), Department of Physical Education and Sports, University of Valencia, Valencia, Spain; 5 Grupo de investigación FISIOTER, Fundación Universitaria María Cano, Medellín, Antioquia, Colombia; University of Valencia, SPAIN

## Abstract

**Introduction:**

Systematic reviews have become increasingly popular among researchers due to their importance in decision-making in health and sports. Only 3% of the reviews are considered decent and clinically useful, and 17% are decent but not useful. Therefore, we aimed to synthesize and critically appraise the evidence of systematic reviews assessing the effect of velocity-based resistance training (VB-RT) on health or athletic performance outcomes in adults and older adults.

**Methods:**

We searched MEDLINE (via Ovid), EMBASE (via Elsevier), Cochrane Database of Systematic Reviews (CDSR) (via Ovid), SPORTDiscus (via EBSCO), and Epistemonikos from inception to January 09, 2024, and updated May 26, 2025, to identify reviews of randomized controlled trials and non-randomized controlled trials investigating the effects of VB-RT on health or athletic performance outcomes in adults and older adults. Two reviewers independently selected the studies, extracted data, and assessed the overall confidence in the results of the included reviews with AMSTAR-2 as ‘High’, ‘Moderate’, ‘Low’, and ‘Critically low’. Descriptive analysis was used to summarize the characteristics of the included systematic reviews. We investigated the degree of overlapping in the reviews.

**Results:**

We included 17 reviews published between 2019 and 2025 in 10 countries, with 8222 participants. Most of the reviews (65%) investigated non-athlete adults. Only 4 (24%) used an formal system to evaluate the certainty of evidence. The degree of overlap in primary studies was moderate (CCA = 7.73%). The overall confidence in the results of 16 reviews (94%) was rated as ‘Critically low’, and only one (6%) was rated as ‘Low’.

**Conclusion:**

Systematic reviews of VB-RT studies often have serious limitations. Authors can improve confidence in the results of future reviews by involving methodologists and statisticians and using a rigorous and transparent system to evaluate the certainty of the evidence. Reviewers should also adhere to the latest standards of conduct and reporting, fostering a more cohesive, precise, and reliable understanding of the VB-RT role in performance and health outcomes.

## 1. Introduction

A systematic review (thereafter reviews) must follow explicit and systematic methods to reduce the risk of bias and produce more reliable findings to inform an evidence-based decision-making context. High-quality reviews are essential in the decision-making context; however, an important prerequisite is that reviews have been based on a sound methodology to avoid bias [1,2]. Reviewing and synthesizing evidence is a powerful tool for patient care [1,2]. Reviews, which use rigorous and transparent approaches to synthesize large amounts of evidence, are commonly used to inform healthcare decisions and policies. Reviews of randomized controlled trials are considered the gold standard for evidence production [1,2]. In 2011, the Institute of Medicine (now the National Academy of Medicine) stated that clinical practice guidelines should be informed by a systematic review of the evidence [2]. This creates a need for conducting more and higher-quality reviews.

Reviews have become increasingly popular among researchers due to their importance in decision-making in health and sports. Between 2004 and 2014, the number of annual review publications increased by 8,000 per year, resulting in an average of 22 reviews published per day [3]. This represents a threefold increase from the number observed in 2004 [3]. An observational study revealed that in 2019, 20,073 reviews were published, averaging over 2,400 per month and 80 per day [4]. The study compared the years 2000 and 2019 and found a 20-fold increase in review publications over the last two decades [4].

The recent surge in the production of reviews has raised some concerns. Only 3% of the reviews are considered decent and clinically useful, while 27% are redundant and unnecessary, 20% are flawed beyond repair, and 17% are decent but not useful [5]. For instance, the World Health Organization (WHO) used twenty-one reviews in their last evidence-based guidelines on physical activity and sedentary behavior [6]. Of these, ten (48%) were rated as having low methodological quality, five (23%) were rated as having critically low quality, and only six (29%) had moderate methodological quality [6]. Seventy-one percent of the reviews used by the WHO to develop evidence-based guidelines have an overall confidence rating of critically low to low [6].

Velocity-based resistance training (VB-RT), an approach that uses velocity to provide objective feedback, estimate strength, develop load-velocity profiles, and enable accurate and objective prescription of RT intensity, volume, and recovery, has gained popularity among resistance training researchers, lecturers, and students in sport science during recent years [7,8]. However, evidence-based guidelines for this exercise modality are lacking, making it difficult to develop recommendations for health or athletic performance. VB-RT has been shown to provide significant health benefits [9,10] and improve performance [11,12]. Nevertheless, the methodological quality of the reviews reporting these findings is unclear. In addition, in our preliminary searches, no formal quality appraisal analysis has been done on VB-RT reviews. In this regard, it is imperative to address this gap in current knowledge. Therefore, this review aimed to synthesize and critically appraise the evidence of systematic reviews assessing the effect of VB-RT on health and athletic performance outcomes in adults and older adults.

## 2. Methods

This systematic review followed the guidance of the Cochrane Handbook [13,14]. Due to the specific reporting checklist being currently under development [15], when it was possible, we reported this review following the Preferred Reporting Items for Systematic Reviews and Meta‐Analyses (PRISMA) checklist [16]. The PRISMA checklist is reported in S1 Table. We have registered the protocol of this review in the Open Science Framework (DOI 10.17605/OSF.IO/PAJCN).

### 2.1. Eligibility criteria

We followed the SDMO framework (S- Studies; D – Data; M – Methods; O- outcome (s)) to guide study selection [17] as follows: 1) we included VB-RT [8] reviews of randomized (RCTs) and non-randomized controlled trials (non-RCTs) [14]; 2) in athletes, non-athletes (as defined by systematic reviewers) adults (≥18 years old) or older adults (60 + years old, as defined by the WHO and United Nations) [18,19]; 3) reported a control group consisting of other interventions with exercise (e.g., traditional resistance training, aerobic training, combined training) or no intervention; 4) that investigated the effects on health [20] or athletic performance outcomes [21] (as defined by the authors of the included reviews). Besides, we applied no language or publication date restrictions. We excluded reviews that included purely observational studies.

### 2.2. Information sources

We conducted a systematic literature search in MEDLINE (via Ovid), EMBASE (via Elsevier), Cochrane Database of Systematic Reviews (CDSR) (via Ovid), Epistemonikos, and SPORTDiscus (via EBSCO) from inception to January 09, 2024, and updated May 26, 2025. Furthermore, we have inspected references to all included reviews for further relevant studies.

One review author searched for ongoing reviews in both the PROSPERO database and the Open Science Framework by using free search terms taken from the main search strategies. The same author searched Google Scholar to capture additional grey literature resources (e.g., Institutional reports, dissertations, theses, and conference abstracts).

### 2.3. Search strategy

See S2 Table.

### 2.4. Selection process

Pairs of review authors selected the studies at title, abstract, and full-text independently. Disagreements were discussed among pairs of researchers, with any outstanding disagreements resolved by an independent third author.

### 2.5. Data management

We exported the literature search results to the Rayyan app [22]. We piloted the eligibility criteria in 10% of the anticipated total sample (title, abstract, and full-text stages).

### 2.6. Data collection process

Pairs of review authors extracted data independently and in duplicate using a standardized electronic form created in Microsoft Forms for this project. We piloted 10% of the total sample of included studies in full-text.

### 2.7. Data items

We extracted the following information: publication year, country, type of review (systematic review with or without meta-analysis), review methodology (aim, inclusion criteria, exclusion criteria, number of studies included/excluded, database search, bias assessment tool, certainty of the evidence approach), characteristics of included studies (design, number of included participants, type of participants, training status, age, sex, and countries), frequency (sessions/week), intensity, volume (set, repetition), length (weeks), progression, supervision, RT implementation equipment (machines with pneumatic resistance, free weights, elastic bands), encoder type, the exercise tested, and setting.

### 2.8. Quality appraisal

A pair of researchers independently assessed the overall confidence in the results of all included reviews using the A Measurement Tool to Assess Systematic Reviews (AMSTAR 2) (21–23) [23]. Each AMSTAR question was rated as yes (clearly done), no (clearly not done), partial yes (partial adherence to the standard), or no meta-analysis conducted (questions 11, 12, and 15). Disagreements were discussed among pairs of researchers, with any outstanding disagreements resolved by a third author.

### 2.9. Managing overlapping systematic reviews

We investigated the degree of overlap and the number of unique studies in each included review [24]. We used Graphical Representation of Overlap for Overviews (GROOVE) [25]. GROOVE provided the number of unique and overlapped primary studies, the overall corrected covered area (CCA), CCA adjusted structural missingness (structural zeros), and the number of nodes with low, moderate, high, and very high overlap [25]. A CCA score of 0–5 indicates a slight overlap, 6–10 moderate, 11–15 high, and **>**15 very high [25].

### 2.10. Synthesis methods

The credibility of each review was reflected in an overall confidence rating, which was determined by an evaluation of non-critical and critical domains (seven critical items: protocol registered before the commencement of the review (item 2), adequacy of the literature search (item 4), justification for excluding individual studies (item 7), risk of bias from individual studies being included in the review (item 9), appropriateness of meta-analytical methods (item 11), consideration of the risk of bias when interpreting the results of the review (item 13), assessment of the presence and likely impact of publication bias (item 15)) [23]. A lack of addressing one or multiple critical domains resulted in a, respectively, low or critically low confidence rating. If no critical flaws were present, the presence of non-critical weaknesses determined whether the review received a high (no weaknesses) or moderate (one or more weaknesses) confidence rating. We used the amstar2Vis [26] package in RStudio to report our results.

We narratively summarized the findings of the included reviews that conducted meta-analyses; however, reviews whose meta-analyses were based solely on within-group pre-post changes without a comparator were not summarized.

## 3. Results

### 3.1. Study selection

**Fig 1:**
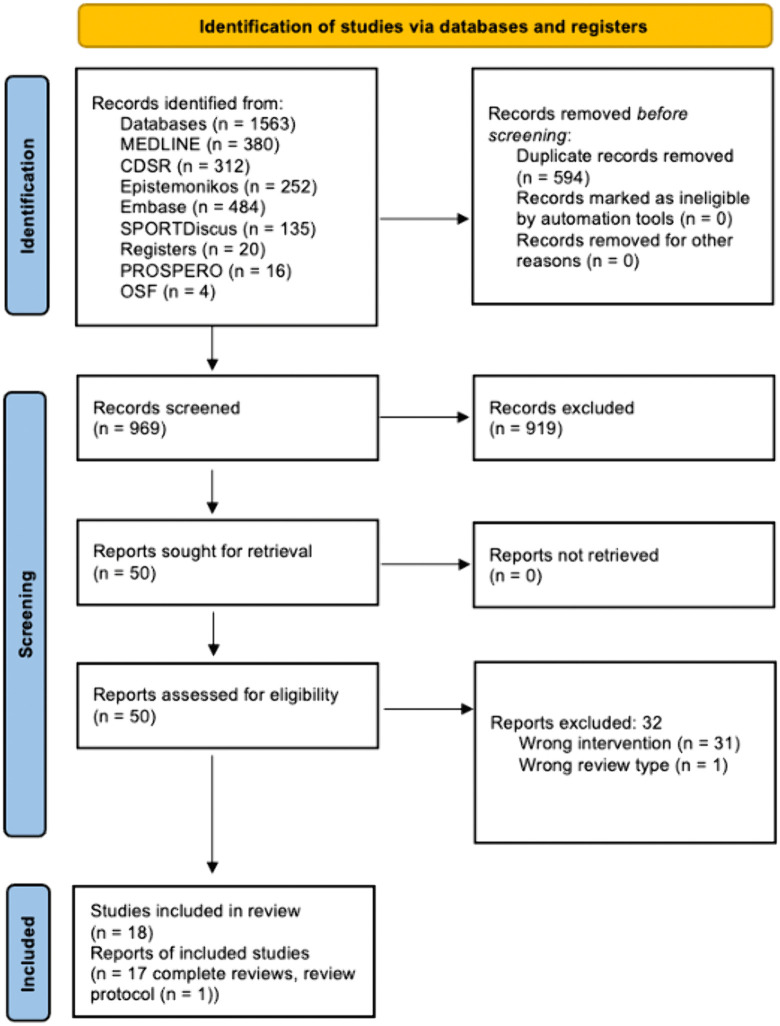
PRISMA flow chart for the literature search.

The initial search identified 1,563 potentially relevant studies. After 594 duplicates were removed, 969 records remained to be screened. We excluded 919 records on title and abstract screening. We assessed 50 full-text articles and excluded 32 full-text reviews (see S3 Table). Eighteen (17 complete reviews [27–43] and one published protocol [44]) published reviews met the inclusion criteria for this review (Fig 1). The characteristics of the included protocol are reported in S4 Table.

### 3.2. Characteristics of the SDMO approach

#### 3.2.1. Type of studies.

**Fig 2:**
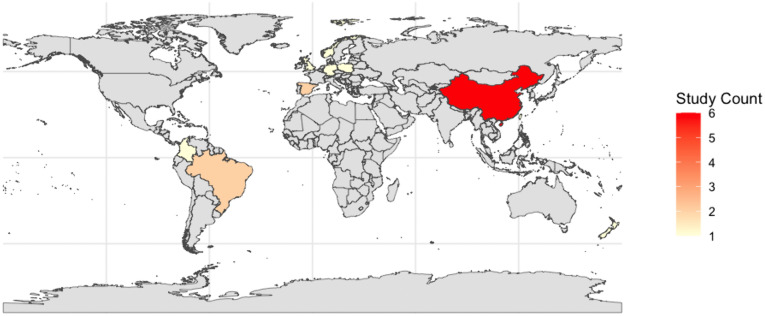
World map with highlighted countries where the 17 included reviews were from (n = 17).

Tables 1 and 2 report the characteristics of the included reviews. Independent research groups conducted all reviews. Ten (59%) out of seventeen reviews did not report the platform used in the screening process [28,30,33,35–37,40–43]. Three reviews have used EndNote [34,38,39], and the remaining (6%, respectively) utilized Rayyan and EndNote [29], Rayyan [32], Mendeley [31], and Excel [27]. 94% [27–35,37–43] (16 reviews) did not search in trial registry repositories, Orange 2022 [36] (6%) searched in ClinicalTrials.gov, ISRCTN, and SportRxiv. The most used risk of bias tool (RoB) was PEDro (6, 35%) [30,31,37,40–42], followed by Cochrane RoB 1 (3, 18%) [28,32,39] and RoB 2 (3, 18%) [27,36,43]. One (6%) review did not report the tool used [33]. Sixteen (94%) out of seventeen reviews described that followed PRISMA to report their systematic reviews [27–33,35–43]. However, fifteen (88%) did not report the PRISMA checklist [27–33,35–42]. Three (18%) out of seventeen reviews did not conduct meta-analyses [27,33,38]. Four (24%) [34,36,37,43] reviews evaluated the certainty of evidence using a formal system (i.e., GRADE), and the remaining 76% (13 reviews) did not rate the certainty of the evidence [27–33,35,38–42]. This body of evidence synthesis was published between 2019 [37] and 2025 [43], and the year with the most reviews published in this field was 2022 (7 reviews, 41%) [27,30,32,36,39,41]. Eleven (65%) out of seventeen reviews included RCTs and non-RCTs [29–35,38,39,41,42]. Six (35%) reviews included only RCTs [27,28,36,37,40,43]. Those RCTs and non-RCTs were published from 1995 to 2024, and China [34,39–43] was the country where most reviews were conducted (6; 35%), followed by Brazil [29,37] and Spain [31,35] with two (12%) reviews, respectively. See Fig 2.

**Table 1:** Characteristics detailed of each included systematic review.

Review ID, year Country	Aim	Number of included trials (N, participants)	Intervention and comparator	Outcomes	RoB and CoE	Overall confidence in the results of the reviews
Baena-Marín 2022 [27] Colombia (https://doi.org/10.6084/m9.figshare.13065950) **Date search:** October 2021	To evaluate the effect of adjusting and controlling loads within a VB-RT program on physical performance variables related to muscular strength and high‐speed actions in trained subjects.	22 (N = 617) Adult athletes	Int: VB-RT Con: PB-RT **Setting:** NR	Muscular strength Sprint performance CMJ jump	Cochrane RoB 2 NR	Critically low
Chen 2024 [28] Taiwan (PROSPERO CRD42021247929) Date search: May 21–22, 2023.	To evaluate the differences in performance-based outcomes between resistance training with low and high VL and the potential moderating effects of different VL schemes on performance-based outcomes.	13 (N = 308) Adults, no Athletes	Int: VB-RT (VL) Con: VB-RT (VL) **Setting:** NR	Muscular strength CMJ jump height HLSV LLSV 20-meter sprint time	Cochrane RoB 1 NR	Critically low
Gantois 2021 [29] Brazil (PROSPERO CRD42020189321) Date search: April 2021	To perform a systematic review of studies that have compared different VL thresholds on muscle strength, physical performance, and muscle hypertrophy during RT; to quantify and compare the findings with a meta-analysis; to draw evidence-based inferences to inform strength-power program prescriptions.	11 (N = 287) Adults, no Athletes	Int: VB-RT (VL) Con: VB-RT (VL) **Setting:** NR	Muscular strength Velocity Outputs at Submaximal Loads (Light and heavy loads) Muscular endurance jump height sprinting time	Modified Cochrane RoB 1 NR	Critically low
Guo 2025 [43] China (PROSPERO CRD42024607528) Date search: September 31, 2024	To examine the existing literature on isokinetic muscle strengthening training for Knee osteoarthritis treatment to provide a more robust evidence-based foundation for clinical rehabilitation practices	33 (N = 2860) Adults, no Athletes	Int: Isokinetic muscle training Con: Other exercise interventions or control (no exercise training) **Setting:** NR	Knee muscle strength Pain Functional scores Knee mobility Physical performance	Cochrane RoB 2 GRADE	Critically low
Held 2022 [30] Germany (NR) Date search: December 6, 2021	To examine and compare the effects of different strength training interventions by distinguishing between traditional and VB-RT approaches based on maximal and explosive performance indices.	13 (N = 311) Adults, no Athletes	Int: VB-RT Con: PB-RT **Setting:** NR	Muscular strength CMJ jump Sprint	PEDro scale NR	Critically low
Hernández-Belmonte 2022 [31] Spain (PROSPERO CRD42021245530) Date search: March 15, 2021	To systematically review the scientific evidence examining the effects of the different VL thresholds during RT on strength and athletic adaptations.	10 (N = 308) Adult Athletes	Int: VB-RT (VL) Con: VB-RT (VL) **Setting:** NR	Muscular strength local muscle endurance Jump capacity Sprint capacity Agility	PEDro scale NR	Critically low
Jukic 2022 [32] New Zealand (OSF: https://osf.io/q4acs/) Date search: June 21, 2022	To synthesize the available evidence on the chronic effects of different VL thresholds on training adaptations.	19 (N = 735) Adults, no Athletes	Int: VB-RT (VL) Con: VB-RT (VL) **Setting:** NR	Muscular strength Muscle hypertrophy Muscle endurance Jump height sprint time COD velocity at a fixed load	Cochrane RoB 1 NR	Critically low
Larsen 2021 [33] (NR) Norway Date search: August 2020	To investigate the effects of subjective and objective autoregulation methods for intensity and volume on enhancing maximal strength during resistance-training interventions.	9 (N = 356) Adults, no Athletes	Int: VB-RT Con: PB-RT **Setting:** NR	Muscular strength	Not reported NR	Critically low
Liao 2021 [34] China (NR) Date search: January 6, 2021	To determine whether VB-RT was more effective than PBT in enhancing strength, jump, linear sprint, and CODs.	6 (N = 124) Adult Athletes	Int: VB-RT Con: PB-RT **Setting:** NR	Muscle strength Load velocity 60%1RM CMJ inear sprint COD	Modified Pedro Scale GRADE	Critically low
Nieto-Acevedo 2023 [35] Spain (PROSPERO CRD42020180911) Date search: November 2022	To examine the differences in the mean propulsive velocities between men and women in the different exercises studied.	6 (N = 249) Adults, no Athletes	Int: MPV men Con: MVP women **Setting:** NR	MPV 30%1RM MPV 70%1RM MPV 90%1RM MPV mean VL	Quality assessment and validity tool for correlational studies NR	Critically low
Orange 2022 [36] United Kingdom (OSF: https://osf.io/pz9fs) Date search: June 1, 2021	To review, meta-analyze, and appraise the quality of evidence regarding the effects of VB-RT vs. traditional resistance training methods on adaptations in strength, power, and linear sprint speed.	4 (N = 88) Adults, no Athletes	Int: VB-RT Con: PB-RT **Setting:** NR	Muscle strength Muscle power Sprint linear speed	Cochrane RoB 2 GRADE	Low
Souza Pontes 2019 [37] Brazil (NR) Date search: June 2018	To analyze the published RCTs that investigated the effects of isokinetic muscle strengthening on muscle strength, mobility, and gait in post-stroke patients.	13 (N = 347)	Int: Isokinetic muscle training Con: Other exercise interventions or control (no exercise training) **Setting:** NR	Muscle strength Mobility Gait speed	PEDro scale GRADE	Critically low
Włodarczyk 2021 [38] Poland (NR) Date search: March 1, 2020	To perform a systematic review of the studies that show the effects of velocity-based resistance training on strength and power performance in elite athletes.	7 (N = 166) Adult Athletes	Int: VB-RT Con: unclear **Setting:** NR	Muscular strength Muscle power	Jaded scale NR	Critically low
Xing Zhang 2023 [40] China (NR) Date search: January 12, 2023	To examine the effect of velocity loss on maximum strength development and to examine the effect of velocity loss on the efficiency of maximum strength development.	9 (N = 336) Adults, no Athletes	Int: VB-RT (VL) Con: VB-RT (VL) **Setting:** NR	Muscular strength MSGPR	PEDro scale NR	Critically low
Xing Zhang 2023 [42] China (NR) Date search: August 2022	To synthesize existing findings concerning velocity loss and training intensity.	27 (N = 693) Adult Athletes	Int: VB-RT (VL) Con: VB-RT (VL) **Setting:** NR	Muscular strength	PEDro scale NR	Critically low
Zhang 2022 [39] China (NR) Date search: April 16, 2021	To evaluate the effects of two resistance training methods on the maximal strength (1RM) of different sports groups through meta-analysis.	10 (N = 184) Adult Athletes	Int: VB-RT Con: PB-RT **Setting:** NR	Muscular strength UB muscle strength LB muscle strength DL and HT muscle strength	Cochrane RoB 1 NR	Critically low
Zhang 2022 [41] China (NR) Date search: 5 September 2021	To examine the effectiveness of VB-RT in enhancing various athletic performances.	9 (N = 253) Adults, no Athletes	Int: VB-RT (VL) Con: VB-RT (VL) **Setting:** NR	Muscular strength MNR CMJ Sprint time	PEDro scale NR	Critically low

**Abbreviation:** CMJ: countermovement jump; COD: change of direction speed; CoE: certainty of evidence; DL: deadlifts; GRADE: grading of recommendations, assessment, development, and evaluation; HT: hip thrust; LB: lower body; HLSV: heavy-load squat velocity; LLSV: light-load squat velocity; MNR: maximum number of repetitions; MPV: mean propulsive velocity; MSGPR: maximum strength gains per repetition; N = number of participants; OSF: open science framework; PB-RT: percentage-based resistance training; RoB: risk of bias; UP: upper body; VL: Velocity loss; VB-RT: velocity-based resistance training.

**Table 2:** Methodological characteristics of systematic reviews.

	Reviews (n = 17)
**Did the review cite a reporting guideline? n (%)**	
PRISMA	16 (94%)
MOOSE	0 (0%)
None	1 (6%)
**Eligible study designs n, %**	
RCTs	6 (35%)
Non-RCTs	0 (0%)
Both	11 (65%)
Databases searched n, %*	
MEDLINE/PubMed	17 (100%)
EMBASE	7 (41%)
Web of Science	12 (71%)
Cochrane CENTRAL	6 (35%)
Scopus	5 (31%)
EBSCO	4 (29%)
SPORTDiscus	7 (41%)
Google Scholar	3 (18%)
Other	14 (82%)
Trial registries searched n, %*	
ClinicalTrials.gov	1 (6%)
ISRCTN	1 (6%)
Other	1 (6%)
**Platforms, software, and/or websites, used for the screening process**	
Rayyan	2 (12%)
EndNote	3 (18%)
Mendeley	1 (6%)
Other	1 (6%)
None	10 (59%)
**Did the review report a replicable search strategy?**	
Yes, the search strategy is replicable	0 (0%)
No, but key terms are reported	10 (59%)
No	7 (41%)
**Did the authors follow any methodological process for prioritizing outcomes?**	
Yes	0 (0%)
no	17 (100%)
**Did the authors use any tool when the meta-analysis was not possible?**	
Yes	0 (0%)
no	17 (100%)
**Method for the synthesis of results**	
Meta-analysis	14 (82%)
Narrative	3 (18%)
Tabular/graphical summary of quantitative results	0 (0%)
**Did the authors report strategies to evaluate heterogeneity?**	
Yes	12 (70%)
No	2 (12%)
Not applicable	3 (18%)
**Did the authors perform a subgroup analysis?**	
Yes	6 (35%)
no	8 (47%)
Not applicable	3 (18%)
**Did the authors of the systematic review perform a sensitivity analysis?**	
Yes	6 (35%)
no	8 (47%)
Not applicable	3 (18%)
**Did the authors use an approach for rating the certainty of the evidence?**	
Yes	4 (23%)
no	13 (76%)

**Abbreviation:** CENTRAL, Cochrane Central Register of Controlled Trials; CINAHL, Cumulative Index to Nursing and Allied Health Literature; MOOSE, Meta-analyses Of Observational Studies in Epidemiology; PRISMA, Preferred Reporting Items for Systematic Reviews and Meta-Analyses.

*Each review can be classified in >1 category.

#### 3.2.2. Type of data.

In total, 8222 participants were included in these reviews. Eleven out of seventeen (65%) reviews investigated non-athlete adults [28–30,32,33,35–37,40,41,43]; the remaining reviews (6, 35%) included adult athletes [27,31,34,38,39,42]. Reviews included studies with participants between 14 [28,32] and 66 [37] years old. In addition, only one (6%) review investigated older adults [37]. Twelve (71%) reviews included studies with females and males [27,28,30,32–39,43], and five (29%) only investigated studies with males [29,31,40–42]. Most of the reviews (9, 56%) included studies with healthy trained participants (i.e., > 1-year experience in resistance training) [28–30,32,33,35,36,40,41], six (35%) investigated primary evidence with athletes [27,31,34,38,39,42], and the remaining (2, 12%) included post-stroke participants [37] and adults who had a diagnosis of Knee osteoarthritis [43].

#### 3.2.3. Comparisons.

The most common comparison was VB-RT against percentage-based resistance training (PB-RT) (6, 35%) [27,30,33,34,36,39] followed by VB-RT with different velocity loss thresholds [31,32,41], with three (18%) reviews (e.g., VL thresholds: 0–10% versus VL thresholds: 20%) and two (12%) reviews compared isokinetic muscle strengthening against other exercise interventions, or control groups [37,43]. Comparing mean propulsive velocity between men and women [35], with one review (6%). Wlodarczyk 2021 [38] did not report the comparison. Zhang 2022 [41], Zhang 2023 [42], and Chen 2023 [28] compared the pre- and post-VB-RT without approach description. Gantois 2021 [29] compared pre- and post-VB-RT of different velocity loss thresholds.

#### 3.2.4. Types of outcome measures.

The most reported outcomes for the reviews were muscular strength (n = 16, 94%) [27–34,36–43], sprint performance (n = 9, 53%) [27–32,34,36,41], vertical jump (n = 8, 47%) [27–32,34,41], and muscle power (n = 2, 12%) [36,38].

### 3.3. Results of Syntheses

#### 3.3.1. Overlapping.

**Fig 3:**
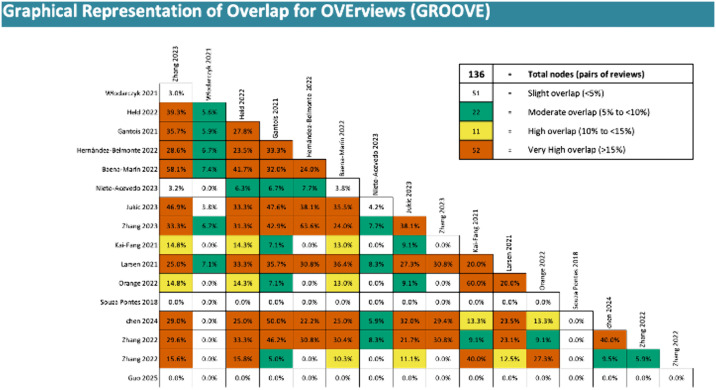
The overall degree of overlapping.

The reviews included a total of 93 primary studies published from 1995 to 2023 (S1 Fig.). After examining the overlap between studies reported in multiple reviews, only 64 unique studies were found. In addition, eight studies were found in two reviews, and 21 other studies were included in three or more reviews. The degree of overlap in primary studies was moderate (CCA = 7.73%). Moderate overlapping was maintained after we adjusted by considering structural missingness (CCA = 8.37%). See Fig 3 and S2 Fig.

#### 3.3.2. Overall confidence in the results of the systematic reviews.

**Fig 4:**
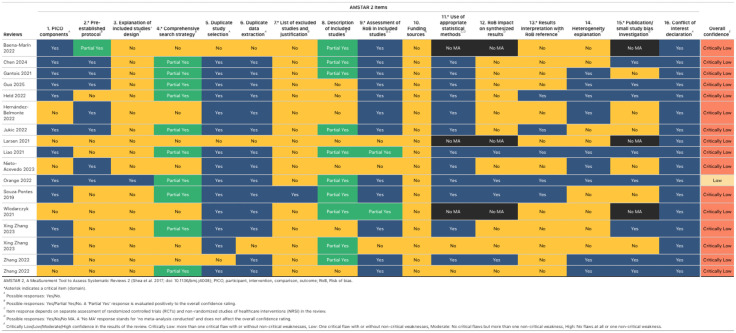
Overall confidence in the results of the reviews (Table).

**Fig 5:**
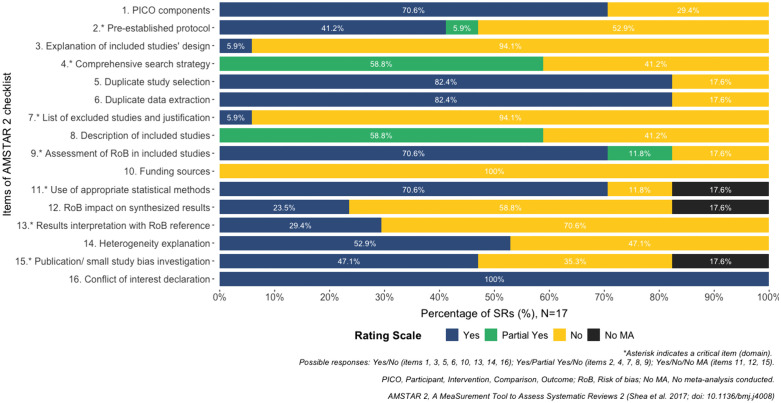
Overall confidence in the results of the reviews (Bar plot).

The results of AMSTAR-2 are shown in Figs 4 and 5, and S3 Fig. The overall confidence in the results of sixteen [27–35,37–43] reviews (94%) was rated as ‘Critically low’, and one (6%) was rated as ‘low’ [36] because most of them did not completely meet the critical domains two (9, 53%) [30,33,34,37–42], four (7, 41%) [27,31,33,35,38,39,42], seven (16, 94%) [27–36,38–43], thirteen (12, 71%) [27–29,31,33,35,38–43], and fifteen (6, 35%) [29,31,32,35,37,42]. That means the reviews have more than one critical flaw and may not be relied on to provide an accurate and comprehensive summary of the available studies addressing the question of interest. Ten reviews (59%) [28–30,32,34,36,37,40,41,43] partially completed a systematic and extensive literature search. In addition, the reviews have not reported the funding source of the primary studies included (100%) [27–43], and 16 [27–35,37–42] out of seventeen reviews did not describe an explanation of the included studies’ design (94%). 10 [28–32,35,40–43] reviews (59%) did not report information about the impact of the RoB on the synthesis results. On the other hand, the domains most covered were any potential source of conflict of interest (17, 100%) [27–43]. Fourteen reviews (82%) reported that more than one author performed study selection [28–32,34–38,40–43] or data extraction [28–32,34–41,43]. 71% (12) of the reviews [27–32,36,37,39–41,43] used a satisfactory method for rating the RoB in the included studies and utilized an appropriate statistical method to pool the results. Besides, all reviews [27–43] have more than one non-critical weakness, which may also diminish confidence in the results of the reviews.

#### 3.3.3. Effectiveness and safety of VB-RT any approach and PB-RT.

A total of 14 reviews conducted a meta-analysis (MA) [28–32,34–37,39–43]. Three reviews with MA [29,41,42] were excluded because the authors meta-analyzed only within-group pre-post mean differences without a comparator (e.g., means pre-VL thresholds: 0–10% versus means post-VL thresholds: 0–10% or means pre-VB-RT any approach versus means post-VB-RT any approach). Four reviews (n = 707 participants) with MA found no difference between VB-RT and PB-RT on muscle strength, jump, sprint, change of direction, and muscle power [30,34,36,39] in athletes and non-athlete adults. When comparing VB-RT with different velocity loss thresholds, four reviews (n = 1687 participants) found little to no difference in muscle strength, jump, sprint, and muscle power in athletes and non-athlete adults [30,34,36,39]. Nieto-Acevedo 2023 [35] found significant differences between men and women in mean propulsive velocity at 30% and 70% and in mean velocities when the participants underwent the mean propulsive velocity method. One review showed a significant difference between the isokinetic muscle strengthening group compared to other exercise or non-exercise control groups in muscle strength, mobility, and gait speed in post-stroke patients [37]. Similar findings were found in one review (n = 2860 participants) comparing the isokinetic muscle strengthening group to other exercise or non-exercise control groups on knee muscle strength, pain, functional scores, knee mobility, and physical performance in patients with knee osteoarthritis [43]. None of the reviews investigated adverse events. S5 Table reports the certainty of the evidence and the directions of the effects.

#### 3.3.4. Certainty of evidence.

Only four reviews [34,36,37,43] evaluated the certainty of evidence using a formal system. The system used was GRADE. Eleven (69%) outcomes were rated as low, two (12%) were moderate, and three (19%) were rated as very low. The most common downgrading domains were risk of bias (limitation in the study design) (3 out of 4 reviews; 75%), inconsistency (2 out of 4 reviews; 50%), and imprecision (3 out of 4; 75%). See S5 Table.

## 4. Discussion

### 4.1. Summary of main results

This systematic review aimed to synthesize and critically appraise the evidence of reviews assessing the effect of VB-RT on health and athletic performance outcomes in adults and older adults. The overall confidence in the results of those reviews was critically low. Several domains need to be improved. Specifically, domain 2 (registration before conducting the review), domain 3 (explanation of the choice of the study design), domain 4 (having a comprehensive search strategy), domain 7 (providing a list of excluded studies), domain 10 (reporting the source of funding), domain 12 (impact of the RoB on the results), and domain 13 (results interpretation with RoB reference). Our findings suggest that these reviews may not be relied on to provide an accurate and comprehensive summary of the available studies addressing the question of interest.

### 4.2. Limitations of the evidence included in the review

There were several flaws across the 16 reviews. Notable areas include a lack of replicable search strategy, the scarce search of ongoing and unpublished trials, and the poor information about how the screening process was done merits careful consideration. Additionally, all the reviews did not prioritize [45–47] or use a core outcome set [48–50] and did not report the utilization of tools for the narrative reporting of the results [51]; in addition, the lack of a formal system to evaluate the certainty of the evidence is reducing the confidence in the findings of these reviews.

### 4.3. Limitations of the review process

This review was planned, registered, conducted, and reported according to the highest methodological standards [14,52,53]. Furthermore, comprehensive systematic searches, as well as the independent and duplicate approach for review selection, data extraction, and quality appraisal processes, are methodological strengths. Moreover, the research team comprised exercise science professionals as well as physiotherapists and experts in evidence synthesis on resistance training. Our well-detailed assessment critical appraisal supports further use of this methodological review for evidence-informed decision-making (i.e., evidence-based guidelines in health and sport). Additionally, we did attempt to control for biases through processes such as no language restrictions to our search, complementing our database literature searches with hand searching, contacting authors for clarification, and for additional information where indicated, although responses were not always obtained. We searched systematic review registries (i.e., PROSPERO and Open Science Framework) to identify unpublished reviews.

### 4.4. Agreements and disagreements with other studies or reviews

Several studies have critically appraised systematic reviews on the effects of exercise training on health outcomes [54–57]. However, none have focused on the overall confidence in the results of the reviews of VB-RT on health and performance outcomes.

Almeida 2019 [56] critically appraised 38 reviews on the benefits and harms of exercise in chronic non-specific low back pain. This review showed that the overall confidence in the results of 28 reviews was rated critically low. Similar to our results, a study found that eighteen out of nineteen included reviews assessed the chronic effect of physical activity on academic achievement in children, and adolescents were evaluated as critically low [54]. Hansford 2022 [55] reported that of twenty-eight reviews that evaluated exercise interventions in health and disease, nineteen of them were appraised between low (11, 39%) and critically low (8, 29%). An overview of reviews [57] conducted by our group showed that just 16% (18 out of 114 reviews) of the reviews investigated the benefits and harms of exercise training on blood pressure reached moderate quality, and the remaining (84%) were rated as low or critically low.

### 4.5. Implications for practice

Our findings indicate that VB-RT reviews often have important methodological limitations, and practitioners, including physiotherapists, sports doctors, athletes, and individuals engaged in recreational resistance training, should be cautious when interpreting and implementing their results. We also encourage university professors and students in related fields (i.e., physical education, sport science) to approach the teaching and learning of VB-RT from a deeply critical perspective, ensuring that routine and clinical practice are aligned with the quality of the best scientific knowledge currently. Despite hypothetical assumptions in a contrary direction, the findings of the included reviews showed that there were no clear advantages of implementing in practice VB-RT in performance (e.g., strength, jump, power, and change of direction) and health outcomes (e.g., mobility) when compared to PB-RT, however, the certainty of the evidence evaluated with a formal system (GRADE) from three reviews [34,36,37] was rated as low to very low (S5 Table). In other words, the true effect is likely to be substantially different from the estimated effect of those reviews.

### 4.6. Implications for research

We need future well-designed and well-reported systematic reviews of interventions investigating the effectiveness and safety of VB-RT in athletes and recreationally resistance training users. These reviews should follow the methodological guidance of the gold standard handbooks, such as the Cochrane handbook [14] or the JBI [58] manual for evidence synthesis. These handbooks explicitly define key standards that must be followed in a systematic review of interventions, including making publicly available early in the project the a priori decisions (i.e., protocol registration), conducting a comprehensive literature search, ideally including an information specialist, transparently reporting the reasons why studies were excluded at the full-text screening stage, appropriately interpreting the review findings, considering the RoB in the individual studies, and, where feasible, investigating the risk of publication bias.

Additionally, they should adhere to international reporting guidelines such as PRISMA-P [52,59] and PRISMA [53]. We suggested to the authors interested in this topic to find some strategies (i.e., improve the research network) to reduce the research waste because the moderate overlapping degree found in this study indicated that most of the reviews published were unnecessary. Finally, we encourage journal editors and peer reviewers to also be mindful of our findings because many of the issues we describe can be addressed at the peer review stage (e.g., lack of registered protocol and adherence to completeness of reporting checklist).

## 5. Conclusion

Systematic reviews of VB-RT studies often have serious limitations. We encourage evidence users of this topic to be mindful of them. Authors can improve confidence in the results of future reviews by planning the review, conducting comprehensive literature searches, including a search for ongoing studies, involving experts in methods and statistics, and using a rigorous and transparent system to evaluate the certainty of the evidence to conclude. To advance the field, reviewers should also adhere to the latest standards of conduct and reporting, fostering a more cohesive, precise, and reliable understanding of the VB-RT role in performance and health outcomes.

## Supporting information

S1 Table PRISMA checklist. (DOCX)

S2 Table Search strategies. (DOCX)

S3 Table Studies excluded at full-text screening. (DOCX)

S4 Table Ongoing studies. (DOCX)

S5 Table Certainty of the evidence and directions of the effects for comparisons. (DOCX)

S1 Fig Number of primary studies by year of publication. (TIF)

S2 Fig Corrected covered area (Adjusted by structural zeros). (TIF)

S3 Fig Overall confidence in the results of the Reviews. (TIF)

## Abbreviations

AMSTAR-2: A Measurement Tool to Assess Systematic Reviews
Non-RCTs: non-randomized controlled trials
PB-RT: Percentage-based resistance training
PRISMA: The Preferred Reporting Items for Systematic Reviews and Meta-Analyses
PRISMA: The Preferred Reporting Items for Systematic Reviews and Meta-Analyses
RCT: Randomized controlled trials
SDMO: Studies, Data, Methods, Outcome (s), Reviews: Systematic review
WHO: the World Health Organization
VB-RT: Velocity-based resistance training.
