# Supplementary material for: A critical appraisal of systematic reviews assessing the effect of chronic velocity-based resistance training on health and athletic performance outcomes: A systematic review
Source: PLoS One. 2026 Feb 18;21(2):e0342992. doi: 10.1371/journal.pone.0342992 (PMC12915968; doi:10.1371/journal.pone.0342992)
Supplement: S2 Table — (DOCX) [file pone.0342992.s002.docx]

## **S2 Table. Search strategies.**

| **Ovid MEDLINE(R) ALL <1946 to January 09, 2024>** | |
| --- | --- |
| 1 | exp Adult/ |
| 2 | Adult.tw. |
| 3 | exp Adults/ |
| 4 | Adults.tw. |
| 5 | exp "Young Adult"/ |
| 6 | Young Adult.tw. |
| 7 | ("Aged, 80" and over).tw. |
| 8 | exp "Oldest Old"/ |
| 9 | Oldest Old.tw. |
| 10 | 1 or 2 or 3 or 4 or 5 or 6 or 7 or 8 or 9 |
| 11 | dynamic resistance training.tw. |
| 12 | exp "resistance training"/ |
| 13 | resistance training.tw. |
| 14 | exp "strength training"/ |
| 15 | strength training.tw. |
| 16 | Strength.tw. |
| 17 | exp "weight lifting"/ |
| 18 | weight lifting.tw. |
| 19 | strengthening program.tw. |
| 20 | strengthen.tw. |
| 21 | strengthened.tw. |
| 22 | 11 or 12 or 13 or 14 or 15 or 16 or 17 or 18 or 19 or 20 or 21 |
| 23 | velocity loss.tw. |
| 24 | barbell velocity.tw. |
| 25 | movement velocity.tw. |
| 26 | mean concentric velocity.tw. |
| 27 | concentric velocity.tw. |
| 28 | speed.tw. |
| 29 | velocity.tw. |
| 30 | vbt.tw. |
| 31 | velocity based.tw. |
| 32 | velocity-based resistance training.tw. |
| 33 | 23 or 24 or 25 or 26 or 27 or 28 or 29 or 30 or 31 or 32 |
| 34 | exp "review, systematic"/ |
| 35 | review, systematic.tw. |
| 36 | exp Meta-Analysis/ |
| 37 | Meta-Analysis.tw. |
| 38 | 34 or 35 or 36 or 37 |
| 39 | 10 and 22 and 33 and 38 |
| **Embase (Elsevier)** | |
| 1 | Adult/exp |
| 2 | Adult:ti,ab |
| 3 | Adults/exp |
| 4 | Adults:ti,ab |
| 5 | 'Young Adult'/exp |
| 6 | 'Young Adult':ti,ab |
| 7 | ('Aged, 80' AND over):ti,ab |
| 8 | 'Oldest Old'/exp |
| 9 | 'Oldest Old':ti,ab |
| 10 | 1 or 2 or 3 or 4 or 5 or 6 or 7 or 8 or 9 |
| 11 | 'dynamic resistance training':ti,ab |
| 12 | 'resistance training'/exp |
| 13 | 'resistance training':ti,ab |
| 14 | 'strength training'/exp |
| 15 | 'strength training':ti,ab |
| 16 | Strength:ti,ab |
| 17 | 'weight lifting'/exp |
| 18 | 'weight lifting':ti,ab |
| 19 | 'strengthening program':ti,ab |
| 20 | strengthen:ti,ab |
| 21 | strengthened:ti,ab |
| 22 | 11 or 12 or 13 or 14 or 15 or 16 or 17 or 18 or 19 or 20 or 21 |
| 23 | 'velocity loss':ti,ab |
| 24 | 'barbell velocity':ti,ab |
| 25 | 'movement velocity':ti,ab |
| 26 | 'mean concentric velocity':ti,ab |
| 27 | 'concentric velocity':ti,ab |
| 28 | speed:ti,ab |
| 29 | velocity:ti,ab |
| 30 | vbt:ti,ab |
| 31 | 'velocity based':ti,ab |
| 32 | 'velocity-based resistance training':ti,ab |
| 33 | 23 or 24 or 25 or 26 or 27 or 28 or 29 or 30 or 31 or 32 |
| 34 | 'review, systematic'/exp |
| 35 | 'review, systematic':ti,ab |
| 36 | Meta-Analysis/exp |
| 37 | Meta-Analysis:ti,ab |
| 38 | 34 or 35 or 36 or 37 |
| 39 | 10 and 22 and 33 and 38 |
| **Cochrane Database of Systematic Reviews (CDSR) (via Ovid)** | |
| 1 | exp Adult/ |
| 2 | Adult.tw. |
| 3 | exp Adults/ |
| 4 | Adults.tw. |
| 5 | exp "Young Adult"/ |
| 6 | Young Adult.tw. |
| 7 | ("Aged, 80" and over).tw. |
| 8 | exp "Oldest Old"/ |
| 9 | Oldest Old.tw. |
| 10 | 1 or 2 or 3 or 4 or 5 or 6 or 7 or 8 or 9 |
| 11 | dynamic resistance training.tw. |
| 12 | exp "resistance training"/ |
| 13 | resistance training.tw. |
| 14 | exp "strength training"/ |
| 15 | strength training.tw. |
| 16 | Strength.tw. |
| 17 | exp "weight lifting"/ |
| 18 | weight lifting.tw. |
| 19 | strengthening program.tw. |
| 20 | strengthen.tw. |
| 21 | strengthened.tw. |
| 22 | 11 or 12 or 13 or 14 or 15 or 16 or 17 or 18 or 19 or 20 or 21 |
| 23 | velocity loss.tw. |
| 24 | barbell velocity.tw. |
| 25 | movement velocity.tw. |
| 26 | mean concentric velocity.tw. |
| 27 | concentric velocity.tw. |
| 28 | speed.tw. |
| 29 | velocity.tw. |
| 30 | vbt.tw. |
| 31 | velocity based.tw. |
| 32 | velocity-based resistance training.tw. |
| 33 | 23 or 24 or 25 or 26 or 27 or 28 or 29 or 30 or 31 or 32 |
| 34 | exp "review, systematic"/ |
| 35 | review, systematic.tw. |
| 36 | exp Meta-Analysis/ |
| 37 | Meta-Analysis.tw. |
| 38 | 34 or 35 or 36 or 37 |
| 39 | 10 and 22 and 33 and 38 |
| **SPORTDiscus (via EBSCO)** | |
| 1 | (MH Adult+) |
| 2 | (TI Adult OR AB Adult) |
| 3 | (MH Adults+) |
| 4 | (TI Adults OR AB Adults) |
| 5 | (MH "Young Adult+") |
| 6 | (TI "Young Adult" OR AB "Young Adult") |
| 7 | ((TI "Aged, 80" OR AB "Aged, 80") AND (TI over OR AB over)) |
| 8 | (MH "Oldest Old+") |
| 9 | (TI "Oldest Old" OR AB "Oldest Old") |
| 10 | 1 or 2 or 3 or 4 or 5 or 6 or 7 or 8 or 9 |
| 11 | (TI "dynamic resistance training" OR AB "dynamic resistance training") |
| 12 | (MH "resistance training+") |
| 13 | (TI "resistance training" OR AB "resistance training") |
| 14 | (MH "strength training+") |
| 15 | (TI "strength training" OR AB "strength training") |
| 16 | (TI Strength OR AB Strength) |
| 17 | (MH "weight lifting+") |
| 18 | (TI "weight lifting" OR AB "weight lifting") |
| 19 | (TI "strengthening program" OR AB "strengthening program") |
| 20 | (TI strengthen OR AB strengthen) |
| 21 | (TI strengthened OR AB strengthened) |
| 22 | 11 or 12 or 13 or 14 or 15 or 16 or 17 or 18 or 19 or 20 or 21 |
| 23 | (TI "velocity loss" OR AB "velocity loss") |
| 24 | (TI "barbell velocity" OR AB "barbell velocity") |
| 25 | (TI "movement velocity" OR AB "movement velocity") |
| 26 | (TI "mean concentric velocity" OR AB "mean concentric velocity") |
| 27 | (TI "concentric velocity" OR AB "concentric velocity") |
| 28 | (TI speed OR AB speed) |
| 29 | (TI velocity OR AB velocity) |
| 30 | (TI vbt OR AB vbt) |
| 31 | (TI "velocity based" OR AB "velocity based") |
| 32 | (TI "velocity-based resistance training" OR AB "velocity-based resistance training") |
| 33 | 23 or 24 or 25 or 26 or 27 or 28 or 29 or 30 or 31 or 32 |
| 34 | (MH "review, systematic+") |
| 35 | (TI "review, systematic" OR AB "review, systematic") |
| 36 | (MH Meta-Analysis+) |
| 37 | (TI Meta-Analysis OR AB Meta-Analysis) |
| 38 | 34 or 35 or 36 or 37 |
| 39 | 10 and 22 and 33 and 38 |
| **Epistemonikos** | |
| 1 | ((title:(Adult) OR abstract:(Adult)) |
| 2 | (title:(Adults) OR abstract:(Adults)) |
| 3 | (title:(Young Adult) OR abstract:(Young Adult)) |
| 4 | (title:(Aged, 80 AND over) OR abstract:(Aged, 80 AND over)) |
| 5 | (title:(Oldest Old) OR abstract:(Oldest Old)) |
| 6 | 1 OR 2 OR 3 OR 4 OR 5 |
| 7 | (title:(dynamic resistance training) OR abstract:(dynamic resistance training)) |
| 8 | (title:(resistance training) OR abstract:(resistance training)) |
| 9 | (title:(strength training) OR abstract:(strength training)) |
| 10 | (title:(Strength) OR abstract:(Strength)) |
| 11 | (title:(weight lifting) OR abstract:(weight lifting)) |
| 12 | (title:(strengthening program) OR abstract:(strengthening program)) |
| 13 | (title:(strengthen) OR abstract:(strengthen)) |
| 14 | (title:(strengthened) OR abstract:(strengthened)) |
| 15 | 7 OR 8 OR 9 OR 10 OR 11 OR 12 OR 13 OR 14 |
| 16 | (title:(velocity loss) OR abstract:(velocity loss)) |
| 17 | (title:(barbell velocity) OR abstract:(barbell velocity)) |
| 18 | (title:(movement velocity) OR abstract:(movement velocity)) |
| 19 | (title:(mean concentric velocity) OR abstract:(mean concentric velocity)) |
| 20 | (title:(concentric velocity) OR abstract:(concentric velocity)) |
| 21 | (title:(speed) OR abstract:(speed)) |
| 22 | (title:(velocity) OR abstract:(velocity)) |
| 23 | (title:(vbt) OR abstract:(vbt)) |
| 24 | (title:(velocity based) OR abstract:(velocity based)) |
| 25 | (title:(velocity-based resistance training) OR abstract:(velocity-based resistance training)) |
| 26 | 16 OR 17 OR 18 OR 19 OR 20 OR 21 OR 22 OR 23 OR 24 OR 25 |
| 27 | (title:(review, systematic) OR abstract:(review, systematic)) |
| 28 | (title:(Meta-Analysis) OR abstract:(Meta-Analysis)) |
| 29 | 27 OR 28 |
| 30 | 6 AND 15 AND 26 AND 29 |
