# Supplementary material for: A critical appraisal of systematic reviews assessing the effect of chronic velocity-based resistance training on health and athletic performance outcomes: A systematic review
Source: PLoS One. 2026 Feb 18;21(2):e0342992. doi: 10.1371/journal.pone.0342992 (PMC12915968; doi:10.1371/journal.pone.0342992)
Supplement: S3 Table — (DOCX) [file pone.0342992.s003.docx]

## **S3 Table. Studies excluded at full-text screening**

| **Review ID** | **Exclusion reason** |
| --- | --- |
| Balachandran 2022 [1] | Wrong intervention |
| Blazevich 2020 [2] | Wrong intervention |
| Ceccato 2013 [3] | Wrong intervention |
| Coelho-Junior 2018 [4] | Wrong intervention |
| Da Rosa Orssatto 2019 [5] | Wrong intervention |
| Orssatto 2020 [6] | Wrong intervention |
| Davies 2017 [7] | Wrong intervention |
| El Hadouchi. 2022 [8] | Wrong intervention |
| Hackett 2018 [9] | Wrong intervention |
| Haque 2024 [10] | Wrong intervention |
| Liu 2010 [11] | Wrong intervention |
| Lopez 2023 [12] | Wrong intervention |
| Martins 2022 [13] | Wrong intervention |
| Morrison 2023 [14] | Wrong intervention |
| Sklivas 2022 [15] | Wrong intervention |
| Stone 2023 [16] | Wrong intervention |
| Tschopp 2011 [17] | Wrong intervention |
| Zeng 2024 [18] | Wrong intervention |
| Yuan 2025 [19] | Wrong intervention |
| Xie 2025 [20] | Wrong intervention |
| Wang 2025 [21] | Wrong intervention |
| Sun 2024 [22] | Wrong intervention |
| Song 2025 [23] | Wrong intervention |
| Radaelli 2025 [24] | Wrong intervention |
| Andreopoulou 2025 [25] | Wrong intervention |
| Chen 2025 [26] | Wrong intervention |
| Cheng 2024 [27] | Wrong intervention |
| Gómez-Redondo 2024 [28] | Wrong intervention |
| Niyazi 2024 [29] | Wrong intervention |
| Lim 2024 [30] | Wrong intervention |
| LeMense 2024 [31] | Wrong review type |
| Da Silva 2024 [32] | Wrong intervention |

### **Reference (S3 Table)**

1. Balachandran A, Steele J, Angielczyk D, Belio M, Schoenfeld B, N Q, et al. Comparison of Power Training vs Traditional Strength Training on Physical Function in Older Adults: A Systematic Review and Meta-analysis. JAMA Netw Open. 2022;5: e2211623. doi:10.1001/jamanetworkopen.2022.11623

2. Blazevich AJ, Wilson CJ, Alcaraz PE, Rubio-Arias JA. Effects of Resistance Training Movement Pattern and Velocity on Isometric Muscular Rate of Force Development: A Systematic Review with Meta-analysis and Meta-regression. Sports Med. 2020;50: 943–963. doi:10.1007/s40279-019-01239-x

3. Ceccato M, Gurjão ALD, Prado AKG, Gallo LH, Jambassi Filho JC, Gobbi S. Weight training, movement velocity and muscle performance: a systematic review. Rev Bras Ativ Fís Saúde. 2013;18. Available: http://www.epistemonikos.org/documents/5072c1164a87646e44e470f0dd6c23242ed7d318

4. Coelho-Junior HJ, Silva Aguiar S, Calvani R, Picca A, de Azevedo Carvalho D, Rodrigues B, et al. Acute and chronic effects of traditional and high-speed resistance training on blood pressure in older adults: A crossover study and systematic review and meta-analysis. Exp Gerontol. 2022;163: 111775. doi:https://dx.doi.org/10.1016/j.exger.2022.111775

5. Da Rosa Orssatto L, De La Rocha Freitas C, Shield A, Silveira Pinto R, Trajano G. Effects of resistance training concentric velocity on older adults’ functional capacity: A systematic review and meta-analysis of randomised trials. Exp Gerontol. 2019;127: 110731. doi:10.1016/j.exger.2019.110731

6. Orssatto LBR, Bezerra ES, Shield AJ, Trajano GS. Is power training effective to produce muscle hypertrophy in older adults? A systematic review and meta-analysis. Appl Physiol Nutr Metab. 2020;45: 1031–1040. doi:10.1139/apnm-2020-0021

7. Davies T, Kuang, K, Orr, R, Halaki M, Hackett, D. Effect of Movement Velocity During Resistance Training on Dynamic Muscular Strength: A Systematic Review and Meta-Analysis. Sports Med Auckl NZ. 2017;47: 1603–1617. doi:10.1007/s40279-017-0676-4

8. El Hadouchi M, Kiers H, De Vries R, Veenhof C, van Dieën J. Effectiveness of power training compared to strength training in older adults: a systematic review and meta-analysis. Eur Rev Aging Phys Act Off J Eur Group Res Elder Phys Act. 2022;19: 18. doi:10.1186/s11556-022-00297-x

9. Hackett, D, Davies T, Orr R, Kuang K, Halaki, M. Effect of movement velocity during resistance training on muscle-specific hypertrophy: A systematic review. Eur J Sport Sci. 2018;18: 1–10. doi:10.1080/17461391.2018.1434563

10. Haque I, Schlacht TZ, Skelton DA. The effects of high velocity resistance training on bone mineral density in older adults: A systematic review. Bone. 2024;179. doi:10.1016/j.bone.2023.116986

11. Liu C, Latham NK. Progressive resistance strength training for improving physical function in older adults. 2010.

12. Lopez P, Rech A, Petropoulou M, Newton RU, Taaffe DR, Galvão DA, et al. Does High-Velocity Resistance Exercise Elicit Greater Physical Function Benefits Than Traditional Resistance Exercise in Older Adults? A Systematic Review and Network Meta-Analysis of 79 Trials. J Gerontol Biol Sci Med Sci. 2023;78: 1471–1482. doi:10.1093/gerona/glac230

13. Martins AD, Fernandes O, Pereira A, Oliveira R, Goñi FDA, Leite NJC, et al. The Effects of High-Speed Resistance Training on Health Outcomes in Independent Older Adults: A Systematic Review and Meta-Analysis. Int J Env Res Public Health. 2022;19. doi:10.3390/ijerph19095390

14. Morrison R, Taylor S, Buckley J, Twist C, Kite C. High-velocity power training has similar effects to traditional resistance training for functional performance in older adults: a systematic review. J Physiother. 2023;69: 148–159. doi:10.1016/j.jphys.2023.05.018

15. Sklivas AB, Robinson LE, Uhl TL, Dupont-Versteegden EE, Mayer KP. Efficacy of power training to improve physical function in individuals diagnosed with frailty and chronic disease: A meta-analysis. Physiol Rep. 2022;10: e15339. doi:https://dx.doi.org/10.14814/phy2.15339

16. Stone WJ, Tolusso DV, Duchette C, Malone G, Dolan A. Eccentric resistance training with neurological conditions: A meta analysis. Gait Posture. 2023;100: 14–26. doi:10.1016/j.gaitpost.2022.11.018

17. Tschopp M, Sattelmayer MK, Hilfiker R. Is power training or conventional resistance training better for function in elderly persons? A meta-analysis. Age Ageing. 2011;40: 549–556. doi:10.1093/ageing/afr005

18. Zeng Z, Ho C, Sit CH, Wong SH, Liao J, Yang Y. Effectiveness of Exercise Intervention on Mobility, Postural Control, and Falls for Older Adults With Mild Cognitive Impairment: A Systematic Review and Network Meta-analysis. Arch Phys Med Rehabil. 2025;106: 781–800. doi:10.1016/j.apmr.2024.12.002

19. Yuan Y, Wang S, Zhou C, Zhang A, Zhang S, Wang Y. Effects of exercise interventions on cognition, physical function and quality of life among older adults with cognitive frailty: A systematic review and meta-analysis. Geriatr Nur (Lond). 2025;62: 96–107. doi:10.1016/j.gerinurse.2025.01.006

20. Xie S, Yuan Y, Wang J, Bai Y, Wang T, Qiu B, et al. Optimal dose and type of exercise improve walking velocity in adults with Parkinson’s disease: a systematic review and Bayesian network meta-analysis. Sci Rep. 2025;15: 2239. doi:10.1038/s41598-025-85456-7

21. Wang D, Pang X, Shen P, Mao D, Song Q. Effectiveness of various exercise types in reducing fall risk among older adults with diabetic peripheral neuropathy: A systematic review and meta-analysis. J Exerc Sci Fit. 2025;23: 157–166. doi:10.1016/j.jesf.2025.03.005

22. Sun R, Wan J, Tang J, Deng Y, Zhang M, Liu C, et al. Effectiveness of resistance training on body composition, muscle strength, and biomarker in sarcopenic older adults: A meta-analysis of randomized controlled trials. Arch Gerontol Geriatr. 2025;128: 105595. doi:10.1016/j.archger.2024.105595

23. Song H, Ge S, Li J, Jiao C, Ran L. Effects of aerobic and resistance training on walking and balance abilities in older adults with Parkinson’s disease: A systematic review and meta-analysis. Mostile G, editor. PLOS ONE. 2025;20: e0314539. doi:10.1371/journal.pone.0314539

24. Radaelli R, Rech A, Molinari T, Markarian AM, Petropoulou M, Granacher U, et al. Effects of Resistance Training Volume on Physical Function, Lean Body Mass and Lower-Body Muscle Hypertrophy and Strength in Older Adults: A Systematic Review and Network Meta-analysis of 151 Randomised Trials. Sports Med. 2025;55: 167–192. doi:10.1007/s40279-024-02123-z

25. Andreopoulou G, Meharry JB, Jagadamma KC, Van Der Linden ML. Physical activity and exercise interventions in adults with cerebral palsy: a systematic review of quantitative and qualitative studies. Disabil Rehabil. 2025;47: 2170–2184. doi:10.1080/09638288.2024.2391568

26. Chen L, Zhou W, Li J, Xu T, Shi Z. Effects of exercise in older adults with osteosarcopenic adiposity: a systematic review and meta-analysis of randomized controlled trials. BMC Musculoskelet Disord. 2025;26: 342. doi:10.1186/s12891-025-08581-4

27. Cheng F, Li N, Yang J, Yang J, Yang W, Ran J, et al. The effect of resistance training on patients with secondary sarcopenia: a systematic review and meta-analysis. Sci Rep. 2024;14: 28784. doi:10.1038/s41598-024-79958-z

28. Gómez-Redondo P, Valenzuela PL, Morales JS, Ara I, Mañas A. Supervised Versus Unsupervised Exercise for the Improvement of Physical Function and Well-Being Outcomes in Older Adults: A Systematic Review and Meta-analysis of Randomized Controlled Trials. Sports Med. 2024;54: 1877–1906. doi:10.1007/s40279-024-02024-1

29. Niyazi A, Mir E, Ghasemi Kahrizsangi N, Mohammad Rahimi N, Fazolahzade Mousavi R, Setayesh S, et al. The effect of functional exercise program on physical functioning in older adults aged 60 years or more: A systematic review and meta-analysis of randomized controlled trials. Geriatr Nur (Lond). 2024;60: 548–559. doi:10.1016/j.gerinurse.2024.10.019

30. Lim H, Jani NDB, Pang WT, Lim ECW. Community-based exercises improve health status in pre-frail older adults: A systematic review with meta-analysis. BMC Geriatr. 2024;24: 589. doi:10.1186/s12877-024-05150-7

31. LeMense AT, Malone GT, Kinderman MA, Fedewa MV, Winchester LJ. Validity of Using the Load-Velocity Relationship to Estimate 1 Repetition Maximum in the Back Squat Exercise: A Systematic Review and Meta-Analysis. J Strength Cond Res. 2024;38: 612–619. doi:10.1519/JSC.0000000000004709

32. Da Silva RSN, Da Silva DS, De Oliveira PC, Waclawovsky G, Schaun MI. Effects of aerobic, resistance and combined training on endothelial function and arterial stiffness in older adults: A systematic review and meta-analysis. Cè E, editor. PLOS ONE. 2024;19: e0308600. doi:10.1371/journal.pone.0308600
