# Supplementary material for: A critical appraisal of systematic reviews assessing the effect of chronic velocity-based resistance training on health and athletic performance outcomes: A systematic review
Source: PLoS One. 2026 Feb 18;21(2):e0342992. doi: 10.1371/journal.pone.0342992 (PMC12915968; doi:10.1371/journal.pone.0342992)
Supplement: S4 Table — (DOCX) [file pone.0342992.s004.docx]

**Supplementary Table 4. Ongoing studies**

| **Review tittle** | Isokinetic exercise for improving knee flexor and extensor muscles (Protocol) |
| --- | --- |
| **Objective** | To assess the effectiveness and safety of isokinetic training of any kind for knee flexion and extension muscles in healthy adults and in adults with neurological, orthopedic, or rheumatological conditions |
| **Methods** | Systematic review of randomized and non-randomized controlled trials |
| **Participants** | Patients (those with neurological, orthopedic or rheumatological diseases) and healthy adults, in any degree of physical condition, who were trained with an isokinetic device. |
| **Intervention** | The use of any device that allows isokinetic contraction (with controlled velocity and accommodative resistance), for concentric  training, eccentric training, or both, for leg flexor and/or extensor  muscles. |
| **Outcome** | - Improvement percentage in peak torque (NM), total work (Joule), endurance and fatigability ratios, average power (Watts), and functional ability (measured with functional tests and questionnaires) - Pain (measured with pain scores e.g. Visual Analogue Score) - Torque-velocity relationship - Muscle cross-sectional area, and composition before and after training (needle biopsies or magnetic resonance imaging) - Activities of muscles enzymes (hexokinase, malate‐dehydrogenase, 3‐hydroxyacyl CoA dehydrogenase and axo glutarate dehydrogenase) - Any reported adverse events |
| **Contact information** | Carolina G Matarazzo  Rehabilitation Center, Israel Hospital Albert Einstein, Av Albert Einstein 627, São Paulo, Morumbi, 05351901, Brazil.  [carolinamatarazzo@terra.com.br](mailto:carolinamatarazzo@terra.com.br) |
| **Notes** | CD005618. DOI: 10.1002/14651858.CD005618. |
