# Supplementary material for: A critical appraisal of systematic reviews assessing the effect of chronic velocity-based resistance training on health and athletic performance outcomes: A systematic review
Source: PLoS One. 2026 Feb 18;21(2):e0342992. doi: 10.1371/journal.pone.0342992 (PMC12915968; doi:10.1371/journal.pone.0342992)
Supplement: S5 Table — (DOCX) [file pone.0342992.s005.docx]

**S5 Table. Certainty of the evidence and directions of the effects for comparisons.**

| **Study ID** | **Intervention** | **Comparison** | **Outcomes** | **Effect estimate direction** | **Certainty of the evidence** | **Confidence in the results of systematic reviews (AMSTAR 2)** |
| --- | --- | --- | --- | --- | --- | --- |
| Held 2022 [30] | VB-RT | PBRT | Muscle strength | = | NA | Critically low |
|  |  |  | Jump | = | NA |  |
|  |  |  | Sprint | = | NA |  |
| Liao 2021 [34] | VB-RT | PBRT | Muscle strength | = | Low | Critically low |
|  |  |  | Load velocity 60%1RM | = | Low |  |
|  |  |  | Countermovement jump | = | Low |  |
|  |  |  | Linear sprint | = | Low |  |
|  |  |  | COD | = | Low |  |
| Orange 2022 [36] | VB-RT | PBRT | Muscle strength | = | Low | Low |
|  |  |  | Muscle power | = | Low |  |
|  |  |  | Sprint linear speed | = | Very low |  |
| Zhang 2022 [39] | VB-RT | PBRT | Muscle strength | + | NA | Critically low |
|  |  |  | UB muscle strength | = | NA |  |
|  |  |  | LB muscle strength | = | NA |  |
|  |  |  | DL and HT muscle strength | = | NA |  |
| Hernández-Belmonte 2022 [31] | VB-RT (Low-mod VL thresholds: ≤25% VL) | VB-RT (mod-high VL thresholds: >25%) | Muscular strength: at moderate/high loads | = | NA | Critically low |
|  |  |  | Muscular strength: at low loads | + | NA |  |
|  |  |  | Muscle strength | = | NA |  |
|  |  |  | muscle endurance | = | NA |  |
|  |  |  | Sprint | = | NA |  |
|  |  |  | Vertical jump | = | NA |  |
| Chen 2024 [28] | VL thresholds: 0-10% | VL thresholds: 20% | Muscle strength | = | NA | Critically low |
|  |  |  | CMJ | = | NA |  |
|  |  |  | HLSV | = | NA |  |
|  |  |  | LLSV | = | NA |  |
|  |  |  | Sprint time | = | NA |  |
|  | VL thresholds: 0-20% | VL thresholds: 30%-45% | Muscle strength | = | NA |  |
|  |  |  | CMJ | = | NA |  |
|  |  |  | HLSV | + | NA |  |
|  |  |  | LLSV | + | NA |  |
|  |  |  | Sprint time | + | NA |  |
| Jukic 2022* [32] | VL thresholds (0% to 55%) | VL thresholds (0% to 55%) | Muscle Strength | = | NA | Critically low |
|  |  |  | Muscle hypertrophy | + | NA |  |
|  |  |  | Muscle endurance | = | NA |  |
|  |  |  | Vertical jump | = | NA |  |
|  |  |  | Sprint time | = | NA |  |
|  |  |  | Velocity against submaximal (low and moderate) Loads | + | NA |  |
| Xing Zhang 2023* [40] | VB-RT with different VL (0% to 50%) | VB-RT with different VL (0% to 50%) | Muscle strength | + | NA | Critically low |
|  |  |  | MSGPR | + | NA |  |
| Nieto-Acevedo 2023 [35] | MPV men | MVP women | MPV 30%1RM | + | NA | Critically low |
|  |  |  | MPV 70%1RM | + | NA |  |
|  |  |  | MPV 90%1RM | = | NA |  |
|  |  |  | MPV mean VL | + | NA |  |
| Souza Pontes 2019 [37] | Isokinetic muscle strengthening | Other exercise interventions or control | Muscle strength | + | Moderate | Critically low |
|  |  |  | Mobility | + | Moderate |  |
|  |  |  | Gait speed | + | Low |  |
| Guo 2025 [43] | Isokinetic muscle strengthening | Other exercise interventions or control | knee muscle strength | + | Low | Critically low |
|  |  |  | Pain | + | Low |  |
|  |  |  | Functional scores | + | Low |  |
|  |  |  | knee mobility | = | Very low |  |
|  |  |  | physical performance | = | Very low |  |
| **Abbreviation:** CMJ: countermovement jump; COD: change of direction speed; DL: deadlifts; HT: hip thrust; LB: lower body; HLSV: heavy-load squat velocity; LLSV: light-load squat velocity; MNR: maximum number of repetitions; MPV: mean propulsive velocity; MSGPR: maximum strength gains per repetition; PBRT: percentage-based resistance training; UP: upper body; VL: Velocity; VB-RT: velocity-based resistance training.  +: Effect estimates to favor interventions.  =: No differences between groups.  -: Effect estimates to favor control.  *: Meta-regression | | | | | | |

It is uncertain whether VB-RT or PB-RT may improve muscle strength, jump, sprint, COD, and muscle power in athletes and trained adults because the quality of the evidence is low to very low [34,36]. Moderate-quality evidence shows that isokinetic muscle strengthening probably improves muscle strength and mobility for post-stroke patients; in addition, isokinetic muscle strengthening may improve gait speed in the same population [37]. It is uncertain whether the isokinetic muscle strengthening group, compared to other exercise or non-exercise control groups, may improve knee muscle strength, pain, functional scores, knee mobility, and physical performance in patients with knee osteoarthritis because the quality of the evidence is low to very [43].
